# Ethnicity and Nonsurgical Rhinoplasty

**DOI:** 10.1093/asjof/ojac035

**Published:** 2022-05-02

**Authors:** Georges Ziade, Ali Mojallal, Mark Ho-Asjoe, Juan Carlos Arenas, Benjamin Ascher

**Affiliations:** Department of Otolaryngology, Head & Neck Surgery, Faculty of Medical Science, Lebanese University, Beirut, Lebanon; Department of Plastic Reconstructive and Aesthetic Surgery, University of Lyon, Lyon, France; Department of Plastic Surgery, St Thomas’ Hospital, London, UK; University of Bogotá D.C., Colombia

## Abstract

**Background:**

Nonsurgical rhinoplasty is a procedure that is gaining popularity in aesthetic clinics particularly because of its minimally invasive nature compared with surgery. It is recognized that there are ethnic variations in nose injection techniques and planned aesthetic outcomes.

**Objectives:**

The objective of this study was to explore experts’ views about the ethnic differences in the anatomical features of the nose and procedure-related considerations in nonsurgical rhinoplasty.

**Methods:**

Using a priori set topics and questions, 4 expert aesthetic physicians, from 4 different ethnic backgrounds and working in 4 different regions, were asked to describe the essential elements to be considered when planning a nonsurgical rhinoplasty, including product choice, injection technique, safety measures, and any practical hints to facilitate achieving the desired outcome.

**Results:**

All invited experts responded to the full set of questions. There were similarities between the treating physicians in some of the technical steps. Nevertheless, there were several differences identified regarding baseline anatomy and patient expectations that could be attributed to ethnicity. Patients’ and physicians’ expectations regarding a successful nonsurgical rhinoplasty can vary depending on their ethnic backgrounds. Therefore, with the current global ethnic and cultural diversities, in addition to the knowledge of the nasal anatomy and safe injection techniques, it is imperative that aesthetic practitioners have full awareness and a good understanding of these ethnic variations.

**Conclusions:**

Nonsurgical rhinoplasty is a highly demanded aesthetic procedure. Patients’ ethnic differences need to be carefully taken into consideration when discussing, planning, and performing nasal fillers injection.

The nose is an important aesthetic unit that has a major impact on each person’s mid-facial beauty and overall appearance. Therefore, surgical rhinoplasty is one of the most commonly performed aesthetic surgeries worldwide. Moreover, it is an important topic for scientific meetings, publications, and training courses.

There have been significant advancements in nasal surgical techniques, but one of the main limitations for patients is the surgical downtime. Therefore, the introduction of nonsurgical rhinoplasty during the last 2 decades gained a lot of popularity among aesthetic physicians and patients. The success of nonsurgical rhinoplasty was assessed with regard to several aspects, among which are the safety of the procedure, significance of the aesthetic change from the practitioner’s and patient’s perspectives, durability of the results, reversibility of undesired changes, and the ease of the procedure as an in-office treatment.^1,2^ The use of hyaluronic acid fillers in rhinoplasty increased the popularity of the procedure, especially with the use of moderate to high volume impact fillers. Furthermore, the subsequent improvement in injection techniques made this procedure one of the most commonly performed procedures in aesthetic clinics.^3,4^

In recent years, the topic of ethnic rhinoplasty became an important subject of discussion, especially in international scientific meetings. Different ethnic populations have shown significant differences in the way they perceive and expect their nose to look like after correction.^5^ Hence, knowing the optimal approach for each ethnic nose is an essential pre-requisite for almost every aesthetic practitioner performing in-office nonsurgical rhinoplasty, especially due to the diversity of ethnicities within each population because of massive population migrations, inter-racial mixings, and the increased frequency of doctors visiting other worldwide destinations to perform aesthetic procedures.^6^ The objective of this study was to explore any ethnic differences in the anatomical features of the nose and the essential elements to be considered when planning and performing a nonsurgical rhinoplasty, including product choice, injection technique, safety measures to be undertaken during the procedure, and any practical hints to facilitate achieving the desired outcome.

## METHODS

The IRB of the Lebanese University approved this study. Written consent was provided, by which the patients agreed to the use and analysis of their data. Four expert aesthetic physicians working in 4 different global regions (expert 1 [G.Z.: the Middle East and Gulf], expert 2 [M.H.-A.: Asia], expert 3 [A.M.: Europe], and expert 4 [J.C.A.: South America]) were asked to answer a series of a priori set questions covering the topics of anatomy and aesthetic considerations; equipment and fillers; procedure, risks, and limitations; and longevity and follow-up (Table 1). The 4 ethnic noses explored in this study were the Arab/Phoenician nose (the Middle East and the Gulf region), Asian nose (Asia), Caucasian nose (Europe), and the Hispanic/Latino nose (South America).

**Table 1.** The A Priori Set Topics and Questions Used in the Study

Topics	Questions
Anatomical and aesthetic considerations	Q1: What are the anatomical characteristics of the specific ethnic nose?
	Q2: What are the aesthetic considerations for the specific ethnic nose?
Equipment and fillers	Q3: What are the filler characteristics you look for?
	Q4: Do you use a needle and/or a cannula or both to inject and why?
	Q5: What is the average amount of filler used in each nasal site?
Procedure	Q6: Describe your step-by-step nonsurgical rhinoplasty technique?
Risks and limitations	Q7: What are the danger zones?
	Q8: What are the aesthetic and anatomical limitations in nonsurgical rhinoplasty?
Longevity and follow-up	Q9: How long do the results last in general?
	Q10: Do you always ask the patient to come back for a touch-up?
	Q11: When is the best time for the follow-up visit?

## RESULTS

Experts’ responses to the pre-set topics and questions in relation to the nose in different ethnic groups and nonsurgical rhinoplasty were as follows:

### Anatomical Characteristics and Aesthetic Considerations

The Arab/Phoenician nose was described as having a high osteocartilaginous hump, wide lower nasal third, minimal to no tip support, and wide alae. People seeking aesthetic correction usually complain of a wide nasal tip that drops down when smiling or talking and an over projected hump on the side view. Therefore, if the alar width is wider than the intercanthal distance on frontal view, even a minimal narrowing can be effective. In contrast, the Asian nose was described as having a straight nasal dorsum, with no hump and a flat overall projection of the nasal bridge. The expert stated that the main concern for most East Asian patients seeking facial aesthetic procedures is the lack of contour on the lateral view, which is accentuated by the flat forehead, no supraorbital ridge, and flat maxillae. Therefore, most Asian patients request looking for a higher nasal bridge to improve the lateral profile and make the nose more defined on the frontal view.

The Caucasian nose was considered to have thin skin, long nasal bones, weak cartilages, a bony hump, and narrow dorsal aesthetic lines with a wide zone at the keystone level, whereas the face of young Hispanic adults, especially females, was described as having a trapezoidal aspect with thick skin, prominent cheekbones and mandibular angles, wide bizygomatic and bigonial diameters, pronounced dental arches, a short retracted-appearing chin, and a tendency toward infraorbital concavity. He stated that the nose tends to be small externally with a broad base and a slightly projected tip, and proposed the view that the requirements of Hispanic women are oriented toward having a refined face with a Caucasian appearance. The expert added that the evaluation of the Latina patient is aimed at defining whether there is a real deficiency at the level of the columella and the nasal tip, taking into account the prominence of the malar structure of the face that generates the false impression of a short columella.

### Equipment and Fillers

For expert 1 (the Middle East and the Gulf region), the preferred filler was one with high G′, unless in case of thin skin or previously operated nose where a medium thickness filler was his preferred option. His most commonly used fillers were Stylage XL/XXL (Vivacy, France) or Restylane Lyft (Galderma, Sweden). In general, the expert stated that a 1-mL syringe was sufficient for a nonsurgical rhinoplasty case (0.2-0.3 mL for the radix, 0.2-0.3 mL for tip support, 0.1 mL for nasal tip prominence, and 0.2 mL for each nasolabial fold triangle). The expert preferred the use of a 29G needle to inject the radix because it provided good control on the injected volume and helped to inject the filler directly on the periosteum, while he preferred the higher safety offered by a 25G cannula when injecting the cartilaginous dorsum, nasal tip, and alar base. If botulinum toxin was to be used, the expert preferred the use of a 33-34G needle because of its better pain profile.

The second expert (Asia) considered Stylage XXL (Vivacy, France) as his filler of choice for nonsurgical rhinoplasty due to its high G′ and low overall swelling. For him, this was particularly useful because, in general, Asian patients are not keen on a wide nasal bridge. On average, the total volume of filler he uses is around 0.8 mL with 0.3 at the radix, 0.3 at the midvault, and 0.2 at the tip. Expert 2 stated that he mainly uses a cannula for injection and only rarely uses a needle for some minor “tiding up,” especially over the radix region.

Expert 3 (Europe) noted that the characteristics of his preferred filler are that, first, it has enough cohesiveness and mechanical force to create a new dorsal shape; second, enough elasticity and rheology to shape the nasal tip; and third, a filler that contains lidocaine. The expert stated that he only uses a needle because it is more precise and easy to use. He suggested that any potential associated risks are significantly reduced with good knowledge of the anatomy. In total, he would use 0.8 to 1 mL (approximately 0.6 mL at the labio-columellar angle, 0.3 mL at the dorsum, and 0.1 mL at the nasal tip). Finally, the fourth expert (South America) expressed that for nose treatment his selection is a filler with a high G′ and a high G″ (high elasticity and high viscosity, respectively) with lidocaine like Stylage XL (Vivacy, France). The expert stated that he uses a needle for all areas and injects the tip with microboluses of 0.025 to 0.1 mL per point and injects 0.05 to 0.2 and 0.2 to 0.5 mL for the dorsum and columella, respectively.

### Procedure

**Figure 1.**
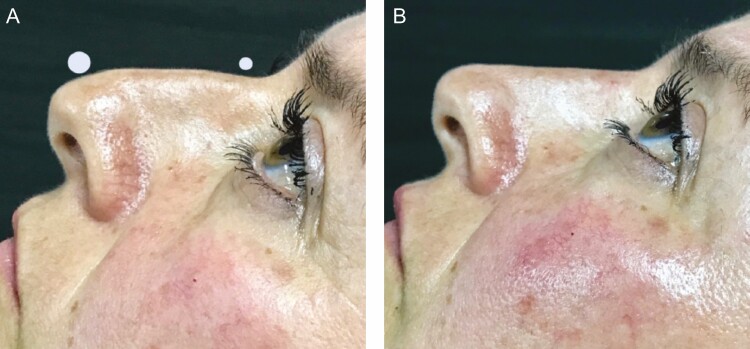
A 45-year-old female patient (A) with nasal bony-cartilaginous hump and droopy nasal tip and (B) 2 weeks after a nonsurgical rhinoplasty using hyaluronic acid fillers.

**Figure 2.**
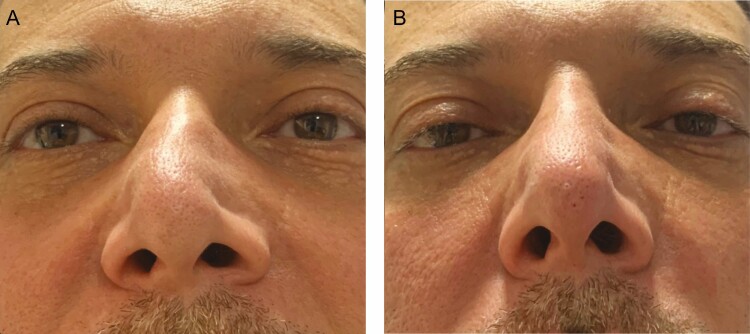
A 45-year-old male patient (A) with an S-shaped nasal dorsum and (B) directly after a nonsurgical rhinoplasty using hyaluronic acid fillers.

**Figure 3.**
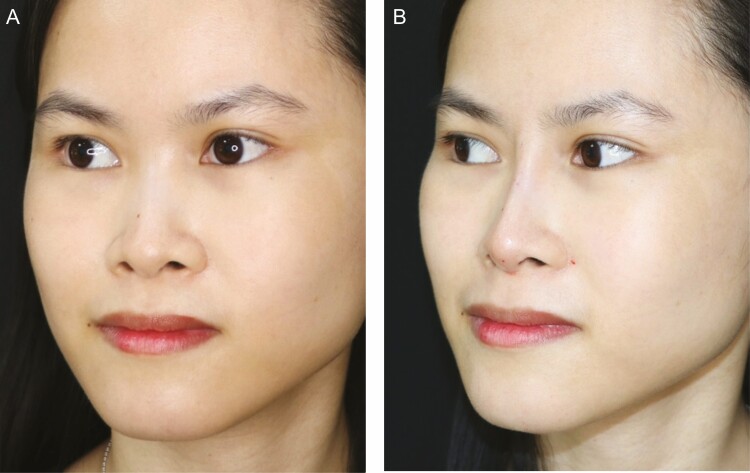
A 28-year-old female patient (A) with a low nasal bony-cartilaginous dorsum and (B) directly after a nonsurgical rhinoplasty using hyaluronic acid fillers.

Apart from listening to the patient’s concerns, understanding their expectations, counseling about the procedure, obtaining a valid informed consent, undertaking baseline aesthetic assessments, and ensuring an aseptic technique, experts were asked to describe, in a step-by-step fashion, their technique for performing a nonsurgical rhinoplasty. These chronological steps are listed in Table 2 (Figures 1-3 and Video).

**Table 2.** Experts’ Responses About Chronology With Which They Perform a Nonsurgical Rhinoplasty

Expert 1 (the Middle East and the Gulf region)	Expert 2 (Asia)	Expert 3 (Europe)	Expert 4 (South America)
- Apply lidocaine 15.6% anesthetic cream at the nasal tip and dorsum and wait for 10 min. - Inject the radix by multiple small boluses using a 29G needle inserted perpendicular to the nasal bone till reaching the desired radix level. - Puncture the nasal tip with a 27G needle at the junction between the upper third and lower two-third of the distance between the nasal tip and nasolabial junction. - Insert a 25G cannula vertically perpendicular to the nasal spine between the 2 medial sides of the lower lateral cartilages injecting while mildly compressing the columella between 2 fingers of the non-injecting hand to support the nasal tip. - Insert the cannula in the subdermis to the point where the nasal tip needs to be most prominent and inject a small bolus of filler. - If indicated, inject a bolus of fillers at the upper nasolabial fold triangle to support the alae using a 27G needle oriented perpendicularly and aspirating before injecting or using a 25G cannula along the nasolabial fold. - If indicated, inject botulinum toxin to the nasalis muscle and depressor septi. - If botulinum toxin is used, the nose is taped to keep the tip supported during the first 5 d.	Retrograde injection starting from the radix downward. Introduction of the cannula through the inferior nasal tip and gliding along the perichondrial plane. It is important not to over inject in the radix as most patients are not looking for a higher nasal take-off point. Over injection in this area will flatten the nasal projection.	A nasal gauze embedded with lidocaine cream and antiseptic is inserted in the nostrils for 5 min and then a 3-step technique is followed: 1. Injection at labiocolumellar angle in 3 incidences to reproduce a columellar strut for opening the labiocolumellar angle and getting more support to the nasal tip. 2. Injection to the upper one-third of the dorsum, between the superior border of the hump and the level of the nasofrontal angle (NFA) to hide the hump and create a straight line from the NFA to the dorsum 3. Injection to the tip of the nose to recreate the tip defining points as a tip graft. Injection by an intranasal approach.	- Cool sense device for anesthesia. - Injection of the selected areas using a 27-30G needle aspirating before injecting. - For the nasal tip, access the transcutaneous infra-tip in superior and anterior directions to the supra-cartilaginous mucosal plane. Possible injection sites are supra-tip, medial tip, or infra-tip. The tip is held between the thumb and index finger at the time of injection. - For the nasal dorsum, supra-periosteal or supra-cartilaginous plane at the upper port and transcutaneous at the osteocartilaginous junction. Microboluses or linear retrograde injection is used. The needle is directed at an angle of 90° in relation to the bone while holding the bony edges between the thumb and index finger. - For the columella, transcutaneous injections are made for definition. Supra-cartilaginous and supra-periosteal injections are used at the levels of the nasal tip and nasal spine, respectively. The injection is performed in a linear retrograde pattern. Columellar injections are performed while holding it between the thumb and index fingers. - Hemostasis by applying pressure or cold compresses. - Injection of botulinum toxin if required. - Monitoring the patient for at least 15 min in search of signs of possible ischemia secondary to treatment and immediate treatment with hyaluronidase if it occurs. - Posttreatment photographs. - Provide general recommendations and warning signs. - Telephone follow-up within 24 h.

### Risks and Limitations of the Procedure

The first expert (the Middle East and the Gulf region) considered any site lateral to the midline a danger zone. He added that injecting the nasolabial triangle using a needle can be considered dangerous if proper aspiration was not done and any injection at the radix level that is not perpendicular to the bone is considered dangerous as well. Similarly, expert 4 (South America) considered any lateral areas to the midline to be risky areas. Expert 2 (Asia) stated that since he uses a cannula and, as long as, he stays in the midline and close to the bony/cartilaginous structure, it should be relatively safe. However, he warned that the operator should avoid over injecting at the nasal tip as the tip support is not particularly strong in an Asian nose, which can result in a tip too big rather than more projecting. The danger zones from the third expert’s opinion (Europe) are the 3 arteries of the nose (columellar artery, lateral nasal artery on the superior part of the alar groove, and the lateral dorsal artery on the lateral side of the dorsum).

With regard to the limitations of the procedure, the first expert (the Middle East and the Gulf region) stated that a high osteocartilaginous hump with a wide nasofrontal angle in a female patient is a limitation to nonsurgical rhinoplasty, because injecting the radix, in this case, may lead to facial masculinization. Furthermore, a wide nasal tip might not benefit from tip support and elevation, while injecting the nasolabial folds triangles in the case of a very wide alar base may be useless, and, in this case, realistic expectations need to be explained to the patient before starting the treatment. For the second expert (Asia), the main limitation of the procedure is if it is used in an Asian nose with a small hump with the aim to achieve a straight dorsum. In this case, it is more difficult to hold the filler around the hump and hence a filler with a high G′ works better. The expert also explained that, in the case of a soft nasal tip, it is always harder to achieve a nice supra-tip break, because in the expert’s opinion, Asians dislike an upturned tip and, because their nose tip is already bulbous, over injection is likely to give a more bulbous tip rather than improved projection. The limitations listed by the third expert (Europe) were a large and wide nose, boxy and wide tip, a long nose with a prominent maxillar spine, and a labio-columellar angle > 105°. Finally, for the last expert (South America), the limitations included the presence of localized bacterial or viral infection, acute or chronic disease at the injection area, history of allergy to hyaluronic acid, previous injection of unknown material, complications associated with previous injections of filling material or previous nasal surgery, and tension causing minimal cutaneous mobility at the level of the nasal dorsum.

### Longevity and Follow-up

From the first expert’s (the Middle East and the Gulf region) point of view, the nonsurgical rhinoplasty effect tends to last 9 to 12 months if the nose is injected for the first time; however, it lasts up to 3 years if injected for the second time, especially at the radix level. The expert stated that he advises his patients to come for an early touch-up 2 to 4 weeks after the initial injection. The second expert (Asia) stated that, on average, the effect lasts around 9 months. He added that he would use touch-up 6 months after the initial injection. In contrast, the third expert (Europe) reported that, in general, no touch-up is needed and that the results last about 1 year in the labio-columellar angle and 16 months in the dorsum and the tip. According to the fourth expert (South America), results of nonsurgical rhinoplasty last 18 to 24 months and he asks his patients to return for touch-up between days 7 and 10.

## DISCUSSION

Nonsurgical rhinoplasty using hyaluronic acid fillers is a commonly performed procedure in current practice and, in many cases, is considered an efficient alternative to surgery because of the minimal downtime. Non-satisfied patients with surgical rhinoplasties often prefer to go for a nonsurgical option for a touch-up in order to prevent longer surgical revision rhinoplasties and all the associated aesthetic and safety issues.^3^

Different injection techniques have been described with regard to the method of injection, its order, and the type of filler used. Some physicians use the top to down approach, and others apply it using the reverse order based on anatomical and aesthetic considerations.^7^ Some authors prefer setting the tip at the right position before working on the dorsum and others prefer straightening the dorsum before lifting the tip to the desired position.^3^

The safety of the procedure was always a concern, and there has been a continuous improvement in the way injectable fillers are delivered to the nose.^8,9^ There are several complications that can happen following nasal injections with fillers—these range from mild ones such as bruising and swelling to very serious ones having a long-term negative impact on the patient’s such as like skin necrosis and vision loss.^10^ Based on the latter, nonsurgical rhinoplasty is considered by physicians as not only a quick and simple but also a delicate office procedure.

At present, ethnic variation is considered an important element to be taken into account when an aesthetic practitioner is performing any procedure.^11,12^ Different ethnic groups perceive the aesthetic determinants of the nose differently, which, in turn, affect their tendency and decision to perform nasal aesthetic surgeries.^5^ As in surgical nose jobs, nonsurgical rhinoplasty presents many differences among different ethnicities, and these differences are essential to be considered by the aesthetic practitioner to provide satisfactory results that can last the longest possible period.^13^

The patient’s perspective of a “nice-looking nose” differs a lot based on their culture and ethnicity, and this needs to be seriously taken into consideration when discussing, planning, and performing the treatment.^14^ Indeed, when addressing the different ethnic noses, a good knowledge and understanding of the patient’s expectations are necessary to plan either an augmentation rhinoplasty, where some structures need to be increased in volume, like doing a camouflage for the nasal hump, and projecting some areas, like creating a midline dome at the nasal tip, or a reduction rhinoplasty, where some hypertrophied muscles that cause nasal enlargement are relaxed using botulinum toxin injection.^15^

Understanding the anatomical and cultural differences regarding the ethnic nose became essential for all aesthetic practitioners, especially due to the diversity in ethnicities present in most of the cities worldwide and the presence of different ethnic characteristics within the same person based on inter-racial marriages.^6,13,16^ In this paper, each one of the 4 experts who is coming from a specific ethnic background described his technique for nonsurgical rhinoplasty. The 4 descriptions had some similar steps, like nasal dorsal hump reduction and tip definition. Nevertheless, the different descriptions showed anatomical variations among different ethnicities, variability in priorities, especially when an increased muscular activity is noticeable, and a non-unified perspective of what constitutes a “beautiful nose.”

However, we appreciate that this descriptive paper has several limitations that can be addressed in future projects: first, the subjective nature of the technical description; second, the lack of the patient’s feedback through a scoring system; third, the lack of an objective tool for assessment; and fourth, the selection of isolated ethnic noses when inter-racial mixing is becoming very common. Nonetheless, the paper still highlights the importance of the topic and demonstrates the need for more research work in this field.

## CONCLUSIONS

Nonsurgical rhinoplasty is considered a safe procedure, which is highly sought after worldwide. A good understanding of anatomy, safety measures, and patients’ expectations is essential to deliver the desired optimal results. Therefore, ethnic differences need to be carefully taken into consideration when performing nasal fillers injection. This paper describes the approach for nonsurgical rhinoplasty in 4 diverse world regions.
